# Potassium at the Origins of Life: Did Biology Emerge from Biotite in Micaceous Clay?

**DOI:** 10.3390/life12020301

**Published:** 2022-02-17

**Authors:** Helen Greenwood Hansma

**Affiliations:** Department of Physics, University of California, Santa Barbara, CA 93106, USA; hhansma@physics.ucsb.edu

**Keywords:** clay, mica, biotite, muscovite, origin of life, abiogenesis, mechanical energy, work, wet-dry cycles

## Abstract

Intracellular potassium concentrations, [K^+^], are high in all types of living cells, but the origins of this K^+^ are unknown. The simplest hypothesis is that life emerged in an environment that was high in K^+^. One such environment is the spaces between the sheets of the clay mineral mica. The best mica for life’s origins is the black mica, biotite, because it has a high content of Mg^++^ and because it has iron in various oxidation states. Life also has many of the characteristics of the environment between mica sheets, giving further support for the possibility that mica was the substrate on and within which life emerged. Here, a scenario for life’s origins is presented, in which the necessary processes and components for life arise in niches between mica sheets; vesicle membranes encapsulate these processes and components; the resulting vesicles fuse, forming protocells; and eventually, all of the necessary components and processes are encapsulated within individual cells, some of which survive to seed the early Earth with life. This paper presents three new foci for the hypothesis of life’s origins between mica sheets: (1) that potassium is essential for life’s origins on Earth; (2) that biotite mica has advantages over muscovite mica; and (3) that micaceous clay is a better environment than isolated mica for life’s origins.

## 1. Introduction

All types of living cells have high intracellular potassium concentrations, [K^+^], to the order of 100 mM [1,2]. When and how did this high [K^+^] appear? This is a mystery [3]. There are two options for when high intracellular [K^+^] might have appeared in living systems: before or after the origins of life. The strongest hypothesis is arguably that life originated in a high-K^+^ environment because maintaining the K^+^ gradient across the cell membrane is energetically expensive [4,5,6,7,8]. The earliest membrane-bound protocells would also have had leaky membranes, causing them to be in equilibrium with extracellular ionic environment [9]. 

As Morowitz and others have noted, features that are ubiquitous in biology are likely to have evolved early in life’s origins [10,11,12]. This also argues for the origin of life in a high [K^+^] environment. Why does life have intracellular K^+^ when it could seemingly use intracellular Na^+^ for the same purpose? Bracher asks [11]. The most logical answer is that life emerged in a high [K^+^] environment. 

In living systems, K^+^-dependent enzymes are typically intracellular, and Na^+^-dependent enzymes are typically extracellular [13,14]. Ribosomes require K^+^ and are essential for life [15]. Many other key cellular processes also require K^+^ [16]. 

Dubina and colleagues propose that life emerged in an environment high in [K^+^], and they showed that potassium ions are better than sodium ions for polymerizing glutamic acid [17,18,19]. Bracher’s group has also researched the advantages of K^+^ during life’s origins [11,20]. For example, K^+^ stabilizes linear dipeptides against hydrolysis, while Na^+^ stabilizes cyclic dipeptides, consistent with the predominance of linear peptides in living systems. 

However, most research on the origins of life ignores potassium K^+^ or only mentions it superficially. The question of K^+^ at life’s origins is, arguably, an elephant in the room of research on the origin of life.

Where was there a prebiotic environment high in [K^+^]? The ocean is not high in [K^+^]. Concentrations of Na^+^ and [Na^+^] are 40 times as high as the [K^+^] concentrations in the ocean. Similarly, river water is not high in [K^+^] [21]. There were K-containing minerals on the early Earth, such as K-feldspars, as well as biotite and muscovite [22]. 

Two main hypotheses have been published for the origins of life in high [K^+^]: in geothermal fields [1] and between the sheets of mica [23], perhaps in micaceous clay. Both options might have been true, especially if micaceous clay was present in geothermal fields. Some advantages of mica are the partial confinement provided by mica sheets and the hexagonal grid of the K^+^ holding mica’s anionic mineral sheets together. This grid has a periodicity of 0.5 nm, which is also the spacing of anionic phosphate groups in extended single-stranded nucleic acids, DNA, and RNA.

Mica provides a non-equilibrium environment for life’s origins due to the temperature changes and water movement that take place between mica sheets. In addition to the sources of energy for life’s emergence in clay, mica provides mechanochemical energy. As Carter and Wills say, living things sustain themselves far from equilibrium by converting a free energy source efficiently into, among other things, mechanical work [24].

## 2. K^+^ between Mica Sheets

**Figure 1:**
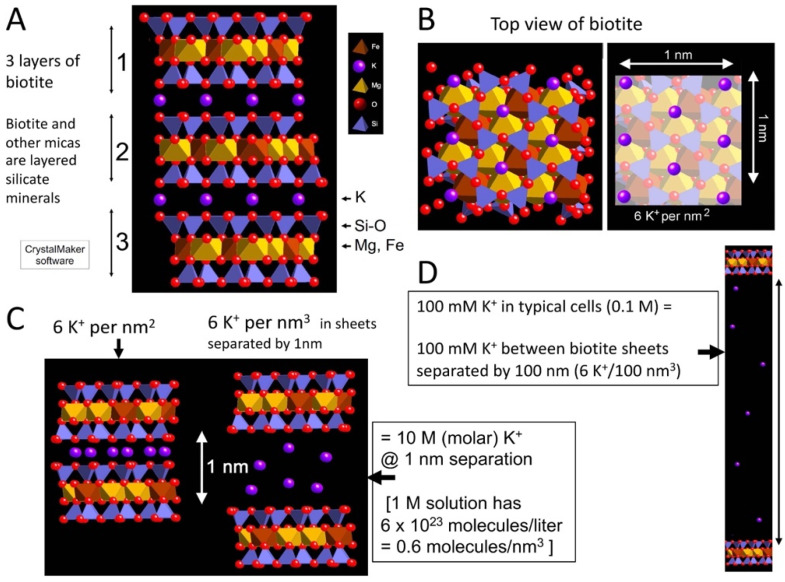
[K^+^] between mica sheets. Structure of the black mica, biotite. (**A**) Side view of three biotite sheets, labeled ‘1’, ‘2’ and ‘3’. (**B**) Top view of 1 nm^2^ biotite, with K^+^ highlighted in the right-hand image, showing that there are six K^+^ per nm^2^ between mica sheets. (**C**) Side view of six mica sheets that are not separated and separated at a distance of 1 nm, where [K^+^] = 10 M between the sheets. (**D**) Scale model of biotite sheets at a separation of 100 nm, where [K^+^] = 100 mM. (CrystalMaker X software, version 10.6.4, CrystalMaker Software Ltd., Oxfordshire, UK).

There are high concentrations of K^+^ between mica sheets (Figure 1). Figure 1A shows three sheets of the black mica, biotite, bridged by K^+^ (purple) between adjacent sheets. K^+^ is at the sites with partial negative charges at the recessed hydroxyl groups on the adjacent sheets.

With an 0.5 nm hexagonal grid of K^+^, there are six K^+^ per nm^2^ between the pairs of mica sheets (Figure 1B), providing a concentration of 10 M K^+^ when the mica sheets are separated by 1 nm (Figure 1C). The sheets need to be separated to a distance of 100 nm to produce a 100 mM concentration of K^+^, which is comparable to the [K^+^] in living cells (Figure 1D). The concentration of 100 mM is the initial [K^+^] concentration when the sheets are separated; the [K^+^] decreases at the edges of the sheets that are in contact with the external environment, and the [K^+^] increases in the inside regions of the split mica, where the sheets are separated by less than 100 nm.

## 3. A Scenario for Life’s Origins between Mica Sheets

**Figure 2:**
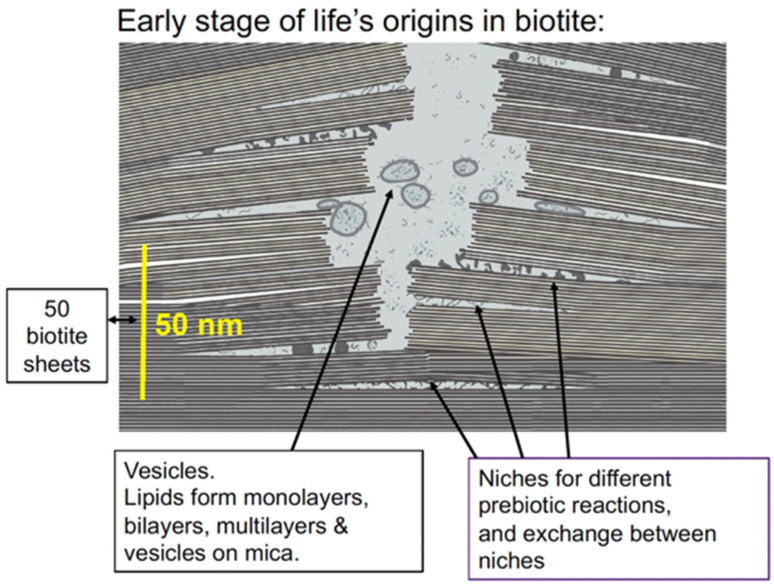
Nanometer-scale diagram of how the early stages of life might have originated between biotite mica sheets. Niches within the biotite sheets provide partially enclosed spaces for the molecular evolution of the different processes that are essential for life. Vesicles form, encapsulating molecules and molecular complexes from the niches.

The spaces between mica sheets provide a semi-enclosed environment for the emergence of life (Figure 2). According to de Duve, in the early stages of life’s origins, the need for free exchanges would have given an advantage to open systems due to the constraints of encapsulation [25]. The spaces between mica sheets have the advantages of open exchanges at the outer edges and also the advantages of partial isolation farther within the spaces between the sheets. This might be ideal, for example, for processes such as the evolution of ribozymes, where isolated niches prevent easily replicated ribozymes from dominating the entire population, and interactions such as ligations can occur in other niches and in the space beyond the sheets, allowing ribozymes to change and evolve [23,26,27].

The niches between biotite sheets could also provide spaces where auto-catalytic cycles and proto-metabolic cycles formed and evolved (e.g., [28,29,30]). In a beautiful piece of work showing the possibilities of prebiotic syntheses, Muchowska et al. synthesized 9 of the 11 main components of the TCA cycle from glyoxylate and pyruvate with Fe(II), (without mica) in a test tube at 70 °C in only hours [29,31]. Vast numbers of niches exist between mica sheets, which also provide spaces for the evolution of genetic coding and ribosomes.

**Figure 3:**
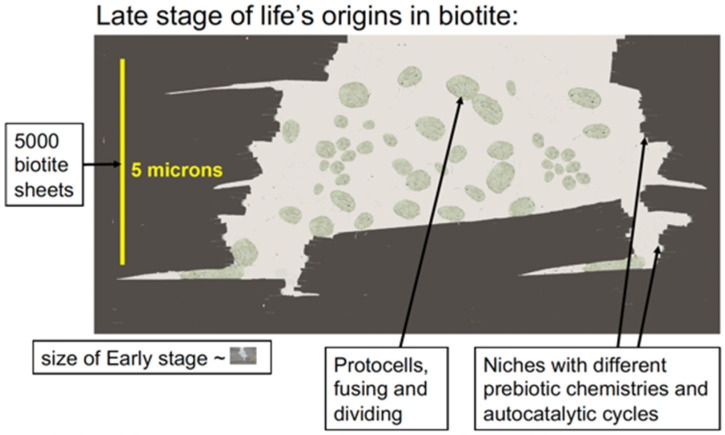
Micron-scale diagram of how life might have originated between biotite mica sheets. Protocells in the aqueous environment encapsulate prebiotic molecular aggregates in the niches between mica sheets. Mechanical energy from moving mica sheets can bleb off protocells, as seen in the lower left corner of the figure. Eventually, a living cell capable of self-reproduction will eventually be produced.

Membranes would form and encapsulate molecular complexes that were accumulating between the biotite sheets, forming vesicles and protocells. These would tend to aggregate and fuse, bringing together the molecular complexes for metabolism, self-replication, protein synthesis, and other necessary processes for life. This would be a slow, gradual, complex process and would occur at many locations in the mica (Figure 3). After a long, long time, these membranes would occasionally encapsulate everything needed for self-reproducing living cells. Some of these living cells would survive, while others would die after a few generations or more. Life indeed emerged on Earth, providing conclusive evidence that some living cells survived.

Mica is old enough to be a site for the origins of life [32]. Muscovite and biotite are among the major minerals found in zircon grains from the Hadean, along with quartz, plagioclase, K-feldspar, chlorite, and hornblende [33]. Most of the mica would not have been in clays as early as the Hadean, but, as Hazen says, even traces of a mineral could have been sufficient for the mineral to be involved in life’s origins [34,35]. Borates, for example, were not present in large quantities at life’s origins [32]. Borates, however, are valuable for stabilizing ribose, and even traces of borate on the early Earth might have served this function [36,37]. Similarly, even traces of micaceous clay might have been the site of life’s origins on Earth.

Biotite mica has advantages over muscovite mica. Biotite is rich in iron (Fe) and magnesium (Mg). The iron is predominately Fe(II) [38]. Especially in a Hadean-reducing environment, Fe(II) predominates over Fe(III). Mg(II) is a major inorganic divalent cation in living systems, where it stabilizes DNA and RNA structures and provides the counterions for ATP, among other things. Biotite is the most conductive mica because of its iron content. Electrical conductivity increases exponentially with the iron content of micas [39,40]. Biotite’s iron may have been useful for redox reactions [41] at life’s origins in the redox-active and conducting environment of clay [42,43] and the reducing atmosphere of the Hadean [44]. Anionic clays such as green rust also catalyze redox reactions [45]. Acid accelerates the dissolution of biotite, acting primarily at step edges of biotite sheets and at etch pits [46]. Biotite is also found on Mars, which may have been the original source of life in the Solar System, seeding life on earth [47]. 

## 4. Energy from Mica

### 4.1. Mechanical Energy

**Figure 4:**
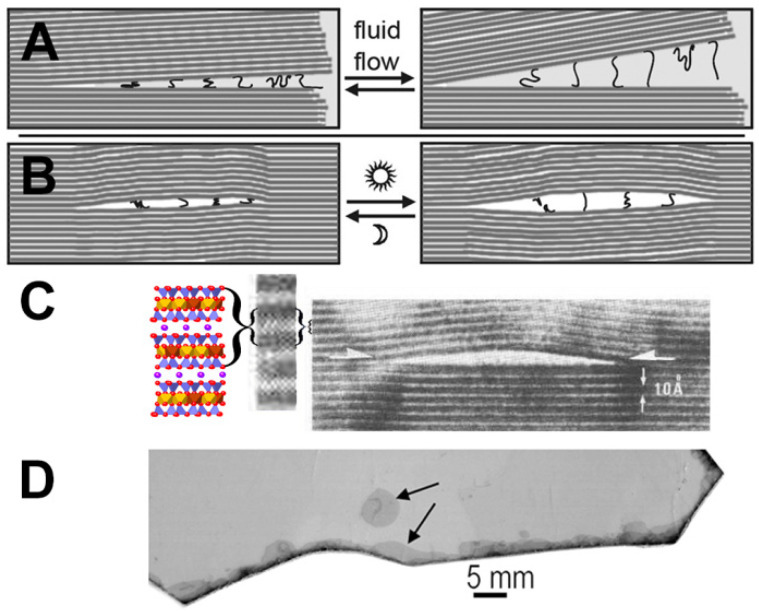
Mica and mechanical energy. (**A**) Diagram of mechanical forces between biotite mica sheets stretching and compressing polymers due to water flow at the edges of the biotite sheets. (**B**) Diagram of mechanical forces between biotite mica sheets due to heat pumps in a biotite bubble. This mechanical energy can be used to synthesize prebiotic molecules, stretch and compress polymers (as shown in the diagram), or bleb off protocells [23]. Seven mica sheets, as shown in (**A**), provide enough force to form a covalent bond in air when moved to a distance of 0.1 nm. (**C**) Biotite bubble (arrows) imaged by HRTEM (high-resolution transmission electron microscopy) [65] with expanded view of HRTEM image and CrystalMaker model of biotite on left. “{“ or “}o” = two biotite layers. The thickness of a single biotite sheet is 1 nm (10 Angstroms). (**D**) Photograph of muscovite mica, showing a bubble (upper arrow) and separation at the edges of the mica sheet (lower arrow). Bubbles are common, even in “high grade” micas such as this one.

Moving mica sheets can produce endless energy for the origins of life (Figure 4) [23,48,49,50]. The energy that is produced by moving mica sheets is mechanical energy that can be used for mechanochemistry, which can be used to make and break chemical bonds when stacks of mica sheets move, open, and shut, in air or water. Mechanochemistry is a growing research field, in which biomolecules are synthesized with mechanical forces [51]. Mechanochemistry has been used in possible prebiotic syntheses [52,53]. Glycine polymerizes by mechanochemistry in mica, which can be achieved by ball milling [54]. Mechanochemistry between mica sheets could happen in either aqueous or dry environments; bubbles in mica sheets, for example, could have provided dry environments (Figure 4B–D).

Both mica sheets and enzymes have open and shut motions that work on the molecules between them. As the title of a recent article says, “Enzymes at work are enzymes in motion” [55]. Experimental results support this statement [56,57,58]. The “fluctuating movements of a motor enzyme” characterize the mechanochemistry of molecular motors [59] and the motions of mica sheets. The mica sheets function as “Mechano-molecular devices” [60]. In an early review of mechanochemistry, bonds were only broken [61]; in reviews published eight years later, there are many examples of syntheses [62], including those of many types of biomolecules [51]. Further evidence for the importance of mechanical energy in biology is that “Molecular Biology of the Cell” is soliciting submissions for its Sixth Special Issue on “Forces on and Within Cells” [63].

How much energy can moving mica sheets provide? If the mica sheets move even 0.1 nanometer (nm) closer together, in air, they can push two molecules together to form a covalent bond if the mica has a spring constant that is stiff enough to provide 170 piconewtons (pN) of force [23]. The equation for a spring constant, F = kx, with x = 0.1 nm, shows that a spring constant (k) of 1.7 N/m (Newtons/meter) is stiff enough. The spring constant of mica depends on the number of mica sheets in the layer that is opening and closing. Each mica sheet is ~1 nm thick. Only about seven mica sheets are needed to provide this spring constant in air [64]. In practice, the layers of moving mica sheets will often be microns, not nanometers, thick, due to the fragility and consequent damage that can be incurred mica sheet layers that are only nanometers thick layers.

Mechanochemical polymerizations can create oligomers and longer polymers that will bind to the mica surface more strongly than monomers and short oligomers. Monomers and short oligomers will be preferentially washed off the mica sheets, favoring polymerization by mechanochemistry over polymer breakdown.

ATP now powers mechanochemistry in cells. How might this transition have occurred if the mechanical energy of the moving mica sheets changed into ATP chemical energy? Sources of chemical energy were evolving during life’s origins. The mechanochemistry of moving mica sheets would become more difficult as the spaces between the mica sheets became clogged with molecules. Hypothetically, a transition to chemical energy could have occurred at some point before the mechanochemistry from mica sheets became too difficult. Many macromolecules and macromolecular processes were developing during the continuum from non-living to living, with the result that the earliest processes would typically be superseded by newer processes before life emerged.

### 4.2. Wet-Dry Cycles

Entropy drives polymerization during wet-dry cycles [66]. Polymers of amino acids and nucleotides form by dehydration, but polymers hydrolyze in the presence of water [67,68,69,70]. In mica, wet-dry cycles occur at split edges of mica sheets, as seen in Figure 4A,D (lower arrow). The slow wet-dry cycles at the edges of mica sheets will generate longer polymers during the longer drying cycles before hydrolysis occurs during the wet phase. These longer polymers will bind to mica better than short polymers and will, consequently, be more likely to remain bound to the surface, as seen in Atomic Force Microscopy (AFM) [71]. In contrast, rapid wet-dry cycles in small particles will cause polymerization to be followed more quickly by depolymerization, resulting in shorter polymers that will be able to detach from the surface more easily. 

How much water was on the Hadean Earth? The question has been reviewed recently, and there is evidence that water may have covered up to 80% of the Earth on the Hadean and more on the Archaean Earth [72].

## 5. How Big Do the Mica Sheets Need to Be?

**Figure 5:**
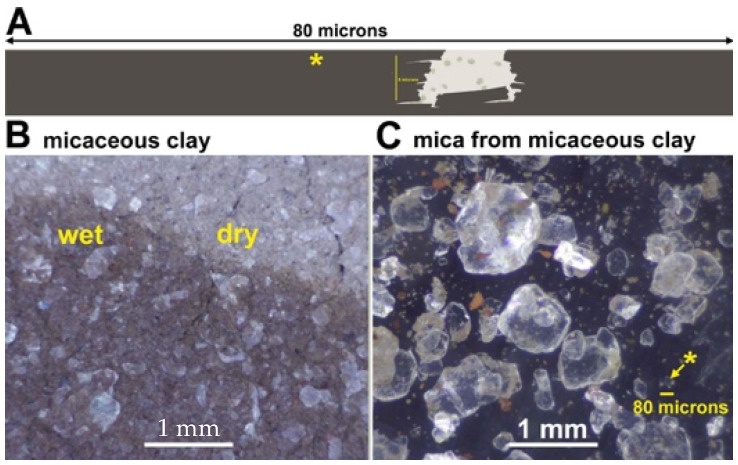
Micaceous clay and the origin of life between mica sheets. (**A**) Mica origins diagram of Figure 3, a late stage in the origin of life, extended to a length of 80 microns. (**B**) Mica Red Micaceous Clay from a New Mexico Clay Store containing pale reflecting pieces of mica in the middle of a wet-dry cycle. (**C**) Mica and a few clay particles washed from the micaceous clay. Yellow asterisk and arrow point to a mica fragment with a diameter of ~80 microns. Yellow asterisks in (**A**,**C**) indicate mica fragments of ~80-microns.

One only needs tiny pieces of mica for mechanochemistry. The mica fragments in micaceous clays are large enough. The “mica world” diagram in Figure 3 is lengthened in Figure 5A to show that even sub-millimeter-sized mica fragments are big enough to generate mechanical energy for life’s emergence. The arrow in Figure 5C points to a submillimeter mica fragment of the same length as the diagram in Figure 5A. Therefore, life may have emerged in micaceous clay as opposed to in larger pieces of mica. The swelling clay particles surrounding the mica fragments would also be advantageous for life’s emergence.

## 6. Clays and the Origins of Life

Why clay? Hyman Hartman explains it as thus:

“The genetic code drives all biological life. But even a mechanism this fundamental rests on still more ancient biochemical processes, as well as the intriguing chemical properties of a seemingly nondescript material—clay. … Formed through the reaction of silicates with water, [cationic] clay minerals have layered crystal structures that provide ideal surfaces for molecules to bind to and interact with each other in close proximity. In fact, we have long used these very properties of clay to speed up chemical reactions in oil refineries and in the catalytic converters found in cars”.

[73]

Many clays have mineral sheets that move apart and together and are “capable of accommodating molecules of any size” [74]. This is one reason for the emphasis on clay as a substrate for the origins of life. Clay minerals can be divided into three categories based on their ion exchange abilities. Cationic clays exchange cations; anionic clays exchange anions; and nonionic clays do not have ion exchange capabilities [75]. Both cationic clays and anionic clays have been regarded as possible sites for the origins of life. [68,74,76,77,78,79,80,81,82]. 

Examples of anionic clays are Layered Double Hydroxides, such as green rust [82]. Anions commonly found in the interlayers of anionic clays are chloride, nitrate, and carbonate [83]. Examples of cationic clays are montmorillonite and micas; typically, Na^+^ is the counterion in montmorillonite, and K^+^ is the counterion in micas; however, there are exceptions.

Are anionic or cationic clays a more likely substrate for life’s origins? Anions such as Cl^−^ that bridge the sheets of cationic clays are generally passive participants in living systems. Arrhenius argues that anions such as phosphate and cyanide are important source components for life and, therefore, that life more likely emerged from anionic clays [74]. The mineral sheets of cationic clays are typically bridged by Na^+^ or K^+^. Living cells expend a great deal of energy maintaining the gradients of Na^+^ and K^+.^ [8]. The importance of the Na^+^ and K+ gradients in living cells provides good evidence that cationic clays were the substrates for life’s origins. The characteristics of living cells are a better indicator of life’s origins than characteristics that might have been useful when life was evolving [12].

## 7. Origin of Life in Micaceous Clay?

**Figure 6:**
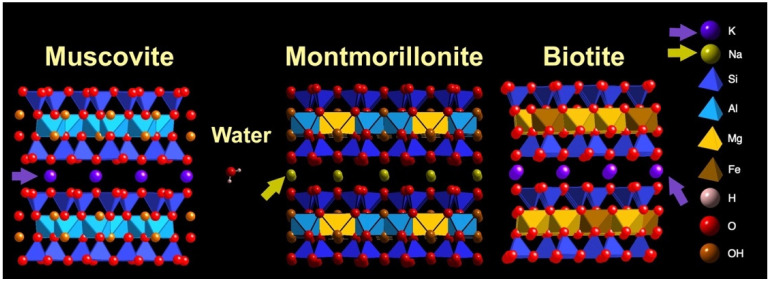
Swelling clay (montmorillonite) and non-swelling clays (muscovite and biotite). Sodium ions, Na^+^, (mustard color) bridge sheets of montmorillonite (mustard-colored arrows); potassium ions, K^+^, (purple) bridge sheets of muscovite and biotite (purple arrows). Ionic radii are 0.095 nm for Na^+^ and 0.133 nm for K^+^ [90]. The smaller Na^+^ are hydrated between montmorillonite sheets, which causes montmorillonite to swell and shrink with wetting and drying. The larger K^+^ between biotite sheets are not hydrated; biotite does not swell and shrink with wetting and drying. Molecular models show two sheets of muscovite mica (left), montmorillonite clay (center), biotite mica (right), and water molecules. Surfaces of the sheets of all three clay crystals are tetrahedral silicon–oxygen (Si-O) layers; see Figure 1B for top view. Clay models and water molecules show relative sizes of atoms and structures. Atom identification list on the right does not show relative sizes of atoms. Atom list = K, Na, Si, Al, Mg, Fe, H, O, OH. (CrystalMaker X software, version 10.6.4, CrystalMaker Software Ltd., Oxfordshire, UK).

Micas and montmorillonite clays have the same mineral structure, with three-layered mineral sheets with T-O-T (Tetrahedral-Octahedral-Tetrahedral) layers and silicon-oxide T layers (Figure 6). Clays such as montmorillonite have been used for the polymerization of amino acids and nucleotides, monomers, e.g., [84]. These polymerizations involved condensing agents such as carbonyl diimidazole or activated monomers, and they have produced oligomers that are up to 55 monomers long. In contrast, muscovite mica has been used for the polymerization of unactivated nucleotides with no condensing agent, producing RNA polymers up to 1000 monomers long [85]. Micas are illite clays; illite is better than other clays at catalyzing the chemical reaction of peroxide with a diamine [86].

Clays such as montmorillonite are “swelling clays” that swell and shrink with wetting and drying cycles [87]. Micas are non-swelling clays, but micas form “books” [88] with frayed edges where there is swelling and shrinking at the sheet edges. Typical clay particles, which are ~1–2 microns in size, are also much smaller than mica particles. Because micas are non-swelling clays, they have a much smaller available surface area than montmorillonite for “test tube” research on clay surface reactivities. For this reason, montmorillonite and other swelling clays have been used for this research, although micas have the same surface reactivities.

The swelling and shrinking of montmorillonite clays is as if they were tiny sandwiches whose filling was growing thicker and thinner as they grow wetter and dryer. Swelling clays have huge surface areas, such that a gram of clay would have the surface area of a tennis court [22]. In contrast, simple measurements show that the surface area of thin (~0.2 mm) mica sheets is ~40 cm^2^ per gram. Non-swelling mica provides a more stable environment for life’s origins than swelling clays. Wet–dry cycles do occur, however, at the split edges of the mica “book”, where water seeps slowly in and out (Figure 4D), leaving dry and nearly dry regions beyond the wet edges. Experiments have shown that water seeped a few millimeters between the sheets of mica pieces that were cycled daily between 22 °C and 4 °C for 2 weeks [23]. 

Why does montmorillonite clay swell while micas do not swell? Both montmorillonite and mica have ion-exchange sites bridging the mineral sheets [22,89]. K^+^ bridges the sheets of micas; Na^+^ bridges the sheets of montmorillonite. Anhydrous K^+^ is larger than anhydrous Na^+^ (Figure 6 caption). The larger ions of K^+^ fill the spaces at the recessed hydroxyls between mica sheets, while the smaller ions of Na^+^ are hydrated at the recessed hydroxyls between the clay sheets, such as montmorillonite. 

Clay mineral surfaces catalyze or support syntheses of amino acids from simple precursors and the polymerizations of amino acids and nucleotides into oligopeptides and oligonucleotides, starting often from activated monomers, e.g., [68,77,78,84,91]. Nucleotides on clay polymerize preferentially in the 3′-5′ orientation, as in life, and not in the non-biological 2′-5′ orientation [92]. 

A Molecular Dynamics study of montmorillonite indicated that mica would have an advantage over montmorillonite because nucleotide polymerization in the 3′-5′ direction occurs the fastest in clay sheets that are closer together compared to in the non-biological 2′-5′direction, which occurs the fastest when the clay sheets experience greater separations [93]. The mineral sheets in mica are more often closer together than the mineral sheets in clay, which swells and shrinks. Polymerizations occur preferentially in the clay interlayer as opposed at to the edges of the sheets. Homochiral polymerizations are favored over achiral polymerizations on clay. Van der Waals forces are the predominant interactions in dry clay, while coulombic interactions are dominant in wet clay [94]. Malani and co-workers have modeled the interactions that mica has with with inorganic cations and water, and they investigated the swelling of mica as a function of cation size [95,96,97,98]. Clays form in association with the water needed for life [99], which is another advantage of clay over some other rocks and minerals.

## 8. Biology and Biotite

Biotite and other micas have similarities with life, as would be expected for places where life might have originated (Table 1) [23,100].

### 8.1. RNA and DNA on Mica

RNA polymers form spontaneously on mica [85]. RNA monomers polymerize non-enzymatically on a mica surface during wet-dry cycles. Nucleotide monophosphates of Adenine (A), Guanine (G), Cytosine (C), and Uracil (U) on mica were cycled through wet-dry cycles at 80 °C and imaged by Atomic Force Microscopy (AFM) [85]. This simple process, with no enzymes or activated nucleotides, produced RNA on bare mica. The RNAs were ~100–1000 nucleotides in length, which is about an order of magnitude longer than the RNA lengths obtained when polymerization occurred in the presence of lipids without mica [102]. It is reasonable that mica’s anionic crystal lattice is a better substrate than lipids for polymerizing RNA because RNA has the same periodicity—0.5 nm—as mica’s crystal lattice.

Mica may have been a template for RNA polymerization at life’s origins. Perhaps nucleic acid linkages are 3′-5′ and not 2′-5′ because the mica sheets served as a template that favored 3′-5′ linkages. Perhaps nucleotide templating on mica’s crystal lattice prevented diphosphate linkages, which form a bent polymer, and other irregularities of nucleotide polymerization.

**Figure 7:**
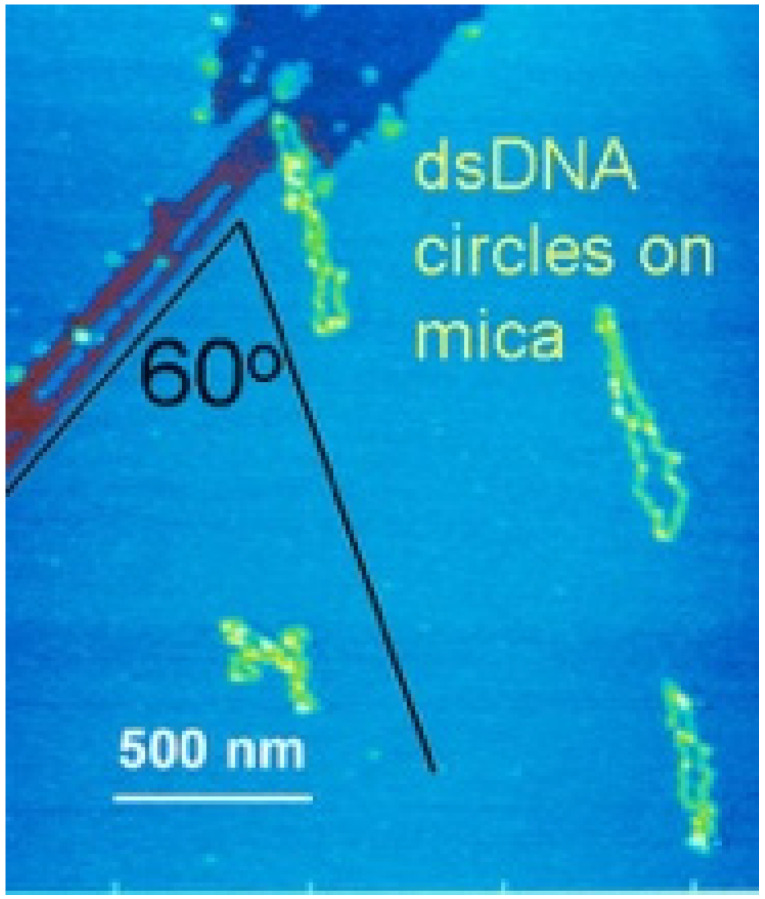
Atomic Force Microscopy of circular double-stranded DNA (dsDNA) on mica with cracks. (Cracks are dark streaks at the upper left). Three of the four dsDNA circles formed a 60^o^ angle with the mica crack, consistent with the alignment on the mica’s hexagonal crystal lattice.

DNA binds reversibly to mica in the presence of various divalent inorganic cations. For example, freshly cleaved mica was soaked in 33 mM magnesium acetate to bind DNA to mica for early AFM imaging (Figure 7) [103,104]. With AFM in aqueous fluid, stable DNA imaging on mica was observed when Ni^++^, Co^++^, and Zn^++^ salts were present; in contrast, DNA binding was not strong enough for AFM imaging when the salts of Mn^++^, Cd^++^, Hg^++^, or K^+^ were used [105]. DNA transcription by RNA polymerase was observed by AFM when Zn^++^ was alternately added to bind the DNA to mica and removed to allow polymerase activity [106].

If polymers have an affinity for a mineral surface, longer polymers will be more firmly bound to the surface than shorter polymers, facilitating the accumulation of long polymers [79]. This has been observed for AFM of DNA [71].

### 8.2. Sugars

Sugars, especially ribose, are a major biomolecule in living systems. A plausible prebiotic reaction for forming sugars is the formose reaction, in which formaldehyde reacts to form sugars [107]. In a test tube, the end products become increasingly large polymers of sugars, branched sugar polymers, and eventually a tarry mess. Monosaccharides, especially ribose for RNA, are a desired product at the origins of life [108]. The spacing of sugars in oligosaccharides is 0.5 nm, similar to the periodicity of the mica lattice.

If the formose reaction is tightly confined between mica sheets, simpler sugars might predominate. Mica’s anionic hexagonal lattice may also favor linear oligosaccharides over branched or bent ones. The formose reaction produces a simple sugar when the reactants are confined in vesicles [109]. This is an example of the advantage that confinement provides for limiting the products of the formose reaction. 

### 8.3. Membranes and the Origins of Life

Membranes on mica have been observed by Atomic Force Microscopy [110,111,112]. Vesicles on mica fuse to form extended bilayers and multilayers. “Lipid worlds” [113,114] could have formed in mica.

Even without lipids, however, mica sheets could have provided partially enclosed spaces for emerging life before the molecules of emerging life were enclosed in membranes. Membranes can be fragile. They leak, acquire and lose molecules, swell, and rupture. The membranes of living cells are highly evolved structures that provide more extensive support and selective permeabilities for their contents than primitive vesicles and membranes.

Lipid membranes might not have been essential at the early stages of the origins of life. Root-Bernstein et al. say that the evolution of membranes would be a late development in their paper about “prebiotic ecology” [115]. An “ecosystems first” perspective was proposed by Baum and others, which was based on their intriguing research involving chemical selection on mineral surfaces [116].

Perhaps, instead of evolving as membrane-bound protocells, protolife evolved as an acellular ecosystem, sharing all the necessary enzymes in an open system. Imagine pieces of this ecosystem periodically being encapsulated in membranes. Nearly all of these membrane-encapsulated protocells would lack some essential component of life or enzymes. Occasionally, membrane-encapsulated protocells would contain all the essential components of life and became alive. Occasionally some of these living protocells would reproduce successfully and begin seeding Earth with life. 

On the other hand, there is also a school of thought in which membranes are the enclosed spaces where proto-life first evolved, e.g., [114]. 

### 8.4. Coacervates and Membraneless Organelles

There is an increasingly popular alternative to membranes at the origins of life—"membraneless organelles” or “membraneless biomolecular condensates”, also known as “coacervates”. Peptides/proteins and RNA interact in membraneless organelles in living cells, such as nucleoli and other particles [117,118,119]. Nucleoli, the membraneless organelles in cell nuclei where ribosomes are formed, are now known to contain other membraneless organelles inside them [120,121]. Membraneless organelles form by liquid-in-liquid phase separation (LLPS) [122]. Membraneless organelles are increasingly of interest to origins-of-life researchers [86,123,124,125,126,127,128] and have been proposed as being the origin of life in mica [129].

Ribosomes are ancient biomolecular condensates that are composed of proteins and RNA and are now necessary for translating nucleic acids into proteins. Ribosomes were present in the Last Universal Common Ancestor of life (LUCA) [130]. When life was coming into being in the pre-LUCA stages, ribosomes and their precursors may have been early “membraneless organelles” that were protected within mica sheets [129]. Prokaryotic ribosomes are ~20 nm in diameter, comparable to the thickness of 20 mica sheets (see Figure 1 and Figure 2) and much smaller than the 100 nm separation of mica sheets at which the [K^+^] is 100 mM (Figure 1D).

## 9. Dielectric Constant at Surfaces

The Dielectric constant, or permissivity, of water is 80 for bulk water but only ~2 for the first two or three water layers above a surface (~2 nm) [131,132]. This means that the charges on charged molecules will become progressively unscreened as the charged molecules approach the mica surface. Electrostatic forces will be stronger, resulting in stronger interactions between charged organic molecules, the anionic mica surfaces, and inorganic cations.

## 10. Crowding at the Origins of Life

“There is a growing consensus that confinement may have facilitated the transformation of inanimate matter into living organisms” [133]. Confinement exists within compartments of various sizes between mica sheets; these compartments may have provided confined spaces for the isolation and stabilization of supramolecular assemblies and protocells.

In cells, molecules are crowded. The space between protein molecules in cells is typically only 10 nm [134]. Crowding speeds up the rates of reactions that are diffusion-limited [135]. Crowding may even be the origin of homochirality [136]. Given the molecular crowding in living cells, molecular crowding at life’s origins is a desirable scenario for hypotheses about the origins of life. Molecules in wet-dry cycles become crowded during the drying phase. Molecules that bind to a surface, e.g., the mica surface, will become concentrated and crowded. Molecules in narrow spaces between mica sheets will typically be crowded by the mica sheets above and below in addition to crowding by other molecules. Clays will also crowd molecules between their sheets, but clay’s swelling will then dilute molecules. Swelling to even two layers of water molecules between Na-montmorillonite clay sheets reduces the interaction energy between the sheets to near zero, according to molecular modeling [94]. Thermophoresis is another way to concentrate molecules in a spatially confined thermal gradient and even to escalate nucleotide polymerization [137,138].

Confinement chemistry would occur between mica sheets during drying and during the compression stage of mechanochemistry. Chemistry in confined spaces produces fewer different molecules and simpler molecules [139,140]. Confined spaces also help proteins fold [141,142]. Enzymes confine their substrates to facilitate the enzymatic reactions. Zeolites mimic enzymes in some respects [143]. Nano-confined liquids have very different properties from bulk liquids [144]. Crowding is also proposed to have enhanced evolutionary capabilities through the networks created by the proximity of components in crowded environments [145]. Confinement chemistry is likely to be a characteristic of any good hypothesis for the origins of life.

## 11. Hierarchy, Complexification, and Error Tolerance

Herbert Simon describes the need for a hierarchy of structures in abiogenesis. [146] He uses the watchmaker as an example. The watchmaker needs stable intermediates in the watchmaking process. If no intermediates were stable, the partially assembled watch would disassemble whenever the watchmaker set it down—to answer the phone, for example, for getting a new order for a watch, to use Herbert Simon’s example. Stable intermediates in abiogenesis can assemble on the hexagonal 0.5 nm anionic grids on and between the “ceilings” and “floors” of the spaces between mica sheets. As Pross and Paschal say, complexification is also an often-ignored but necessary aspect of abiogenesis [147]. Molecular interactions with mica sheets will stabilize intermediates and enable further complexifications.

Freeman Dyson says error tolerance is essential for life’s origins [148]. With the redundancy of the vast areas between biotite mica sheets, in micaceous clay, almost everything can go wrong, and life can still emerge. If not from micaceous clay, life emerged from some other habitat with a vast error tolerance.

## 12. Conclusions

Somewhere in the universe, there was a hospitable habitat that had everything needed for the origins of life. We know this because life now exists on Earth. The habitat may have been on Earth, ~4 billion years ago [149]. In this habitat, the components and processes of life were evolving, eventually resulting in LUCA, a Last Universal Common Ancestor [150,151], which may have been a primitive acellular community of some type [115,116,152,153]. Among these components and processes, RNA-peptide “worlds” [154,155,156,157,158,159,160,161,162] evolved to create and replicate genetic information [163]; proto-metabolic cycles evolved into early metabolism [164,165], and ribosomes evolved to synthesize proteins [166,167]. This may have occurred in “lipid worlds” [102,113,168], which could have been in mica; hydrothermal vents have also been proposed [169,170,171,172]. Vast numbers of interconnections were needed to bring the precursors to Earth or to synthesize them on Earth, eventually bringing them to the hospitable habitat from which “biology” emerged. The habitat was likely clay [76,173], the stuff of life.

Biology may have emerged from the spaces between biotite sheets in micaceous clay. The spaces between the sheets in these mica “books” might have been ancient pre-cellular habitats where prebiotic molecules were confined, concentrated, and synthesized before membrane-bound cells emerged (Figure 2) [23,50,100,129,136]. These pre-cellular spaces have an anionic crystal lattice with “ceilings” and “floors” that could have templated molecular syntheses and polymerizations. The mineral sheets of biotite and muscovite micas have a layered silicate structure, similar to montmorillonite and other clays, which have been used successfully to catalyze reactions non-enzymatically, such as in the synthesis of biopolymers. [77,78,86]. Mica sheets move, open, and closed at their edges as fluids flow and temperatures change; they are thus in a constant state of the thermodynamic non-equilibrium that is necessary for life. 

We will never know for certain whether this or any origins hypothesis is completely true. The experimental method traditionally starts with a hypothesis, followed by experimentally testing the hypothesis. Testable hypotheses are presented here. Experimental results will show what is possible today, but these results may, in fact, be false positives or false negatives. The origins of life are partly an ahistorical science in which much will remain hypothetical, both in terms of experiments and ideas [147]. Experimental results give us ideas about how life might have originated, but they cannot absolutely prove how life originated, though strong experimental results are seen as convincing evidence [31].

## Figures and Tables

**Table 1:** Characteristics of life and mica.

Life:	Mica:
Cellular compartments	Compartments between mica sheets
High intracellular potassium, [K^+^]	High [K^+^]; potassium ions bridge mica sheets
Nucleotides polymerize to DNA and RNA	Nucleotides polymerize to RNA in wet/dry cycles [85]
0.5 nm spacing of anionic phosphates in ssDNA	0.5 nm anionic crystal lattice on mica surface
Exchangeable inorganic cations bridge anionic sites on molecules such as DNA	Exchangeable inorganic cations bridge anionic sites between mica sheets
Water-rich; aqueous	Hydrophilic
Forms H-bonds	Forms H-bonds [101]
Mechanical energy of enzyme motion ^1^	Mechanical energy from moving mica sheets ^1^
Synthesis of biomolecules in confined spaces	Supports chemistry of confinement
Filled and covered with lipid membranes	Supports lipid membranes & vesicles

^1^ The mechanical energy of enzymes is powered by chemical energy, primarily ATP. The mechanical energy of moving mica sheets is primarily powered by thermal disequilibria.

## Data Availability

Not applicable.
