# Correction: Hansma, H.G. Potassium at the Origins of Life: Did Biology Emerge from Biotite in Micaceous Clay? *Life* 2022, *12*, 301

**DOI:** 10.3390/life12050744

**Published:** 2022-05-17

**Authors:** Helen Greenwood Hansma

**Affiliations:** Department of Physics, University of California, Santa Barbara, CA 93106, USA; hhansma@physics.ucsb.edu

The author wishes to make the following correction to this paper [1]:

The No. 66 reference should be corrected as the following one:

66. Ross, D.; Deamer, D. Prebiotic Oligomer Assembly: What Was the Energy Source? *Astrobiology* **2019**, *19*, 517–521.

The author apologizes for any inconvenience caused and states that the scientific conclusions are unaffected. The original article has been updated.
